# Rapid detection of respiratory organisms with FilmArray respiratory panel and its impact on clinical decisions in Shanghai, China, 2016‐2018

**DOI:** 10.1111/irv.12701

**Published:** 2019-12-01

**Authors:** Yiyi Qian, Jingwen Ai, Jing Wu, Shenglei Yu, Peng Cui, Yan Gao, Jialin Jin, Xinhua Weng, Wenhong Zhang

**Affiliations:** ^1^ Department of Infectious Diseases Huashan Hospital Fudan University Shanghai China; ^2^ State Key Laboratory of Genetic Engineering School of Life Science Fudan University Shanghai China; ^3^ National Clinical Research Center for Aging and Medicine Huashan Hospital Fudan University Shanghai China; ^4^ Key Laboratory of Medical Molecular Virology (MOE/MOH) and Institutes of Biomedical Sciences Shanghai Medical College Fudan University Shanghai China

**Keywords:** antibiotic use, clinical decision, FilmArray respiratory panel, respiratory virus, unexplained pneumonia

## Abstract

**Background:**

In this study, we evaluated the diagnostic potential and clinical impact of an automated multiplex PCR platform (the FilmArray Respiratory Panel; FA‐RP), specially designed for pathogen detection in respiratory tract infections in adults with unexplained pneumonia (UP).

**Methods:**

A total of 112 UP patients in Shanghai, China, were enrolled prospectively and assessed using the FA‐RP from October 2016 to March 2018. We examined the test results and their influence on clinical decisions. Furthermore, as a control group, we retrospectively obtained the clinical data of 70 UP patients between October 2014 and March 2016 (before the FA‐RP was available). The two patient groups were compared with respect to factors, including general antimicrobial use and defined daily dose (DDD) numbers.

**Results:**

Between October 2016 and March 2018, the positive rate obtained using FA‐RP for UP was 76.8%. The primary pathogens in adults with UP were Influenza A/B (47.3%, 53/112). Compared with the patients before FA‐RP was available, patients who underwent FA‐RP testing had higher rates of antiviral drug use and antibiotic de‐escalation during clinical treatment. FA‐RP significantly decreased the total DDDs of antibiotic or antifungal drugs DDDs by 7 days after admission (10.6 ± 2.5 vs 14.1 ± 8.8, *P* < .01).

**Conclusions:**

The FA‐RP is a rapid and sensitive nucleic acid amplification test method for UP diagnosis in adults. The application of FA‐RP may lead to a more accurately targeted antimicrobial treatment and reduced use of antibiotic/antifungal drugs.

## 1 INTRODUCTION

After the severe acute respiratory syndrome outbreak, in order to track similarly highly contagious and severe lower respiratory illness with pneumonia symptoms, the Chinese government has paid great attention to unexplained pneumonia (UP), a phrase coined by the Chinese Human Unexplained Pneumonia Surveillance Network (CHUPSN) and published in 2004. UP refers to pneumonia, which could be life‐threatening, characterized by rapid progression, a normal white blood cell count range, and poor response to antibiotics.^1^ Although the common pathogens causing UP are viruses or atypical pathogens, their overlapping clinical manifestations can impede the ability of clinicians to directly diagnose the causative pathogens, which may lead to unnecessary antimicrobial usage. Thus, rapid and precise diagnosis of the causative agents is critical for the prompt management of UP.

To date, culture‐based and other traditional methods have been used to help the clinical approach to diagnose UP; however, these methods have certain disadvantages. For example, rapid antigen testing has only a 40%‐100% sensitivity for adult influenza and is dependent on the use of specific scientific techniques, whereas culturing viruses can take up to 10 days.^2^ In recent years, there has been an increase in the use of nucleic acid amplification test (NAAT)–based methods for the detection of viral pathogens due to their excellent diagnostic ability in identifying a broad spectrum of pathogens.^3^ FilmArray multiplex PCR (BioFire Diagnostics, Inc, a bioMérieux Company) is a NAAT method that can be used to detect multiple pathogens in a single test. In May 2011, the FilmArray respiratory panel (FA‐RP) was FDA cleared for the detection of 17 respiratory viral pathogens, including Influenza A (H1‐2009, H3, H1, and “not subtyped”)/B, respiratory syncytial virus (RSV), human metapneumovirus, adenovirus, coronavirus (HKU1, OC43, NL63, and 229E), human rhinovirus/enterovirus, parainfluenza (including subtype 1, 2, 3, and 4), and three bacteria (*Bordetella pertussis*, *Chlamydophila pneumoniae*, and *Mycoplasma pneumoniae*). The turnaround time using this panel is approximately 1 hour; thus, this technique can ease the rapid and accurate identification of the pathogens causing UP.^4^

With the increasing implementation of the FA‐RP in clinical microbiology laboratories, further studies are required to determine the clinical diagnostic ability of the method and its influence on patient outcomes and antimicrobial usage. Accordingly, in this study, we aimed to evaluate the clinical impact of FA‐RP on UP patient treatment compared with conventional methods. To this end, we prospectively enrolled patients diagnosed with UP and analyzed the pathogen detection ability of the FA‐RP. Moreover, we evaluated the clinical impact of FA‐RP on patients based on a comparison of the clinical outcomes and medical records of the patient cohort enrolled during the period from October 2016 to March 2018 with the data of a cohort retrospectively collected for the period between October 2014 and March 2016. We aimed to evaluate the effect of using the FilmArray in terms of the quantification of antimicrobial use, use of antiviral drugs, and de‐escalation or adjustment of antibiotic treatment.

## 2 MATERIALS AND METHODS

### 2.1 Study design and participants

The prospective cohort was enrolled in Shanghai, China, between October 2016 and March 2018, including two consecutive winter seasons. Eligible patients were recruited based on a modified definition of UP by CHUPSN as follows: (a) fever (>38°C); (b) pneumonia radiographic results; (c) normal or slightly decreased number of total white blood cells in the early stage of the disease; (d) duration of illness of ≤14 days; (e) poor response to initial antibiotic treatment; and (f) a minimum age of 18 years old. Samples from all enrolled patients were submitted to FA‐RP analyses and were accordingly referred to as the “FA‐RP group.” We recorded the demographic characteristics of patients and their relevant clinical data. Patients in the FA‐RP group who were not lost to follow‐up and had complete data were referred to as the “intervention group.”

A historical control cohort was retrospectively recruited using the same inclusion criteria among patients who were hospitalized between October 2014 and March 2016, during which time the FA‐RP was not available. Similar to the FA‐RP group, relevant data of the patients within the control cohort were recorded.

### 2.2 Sampling

Within 24 hours of patient admission, respiratory tract specimens were collected, including bronchoalveolar lavage fluid (BALF: among those patients who gave consent to undergo bronchoscopy), sputum (if expectoration was evident), and nasopharyngeal swab (NPS, if no bronchoscopy was performed or no expectoration sample was collected). Notably, lower respiratory tract specimens were given higher priority (BALF > sputum > NPS) during the study. As soon as possible after sampling, the specimens were transferred to the Laboratory of Infectious Diseases Research Institute, Huashan Hospital, Shanghai.

Professional pulmonary physicians oversaw BALF sampling, while the collection of NPS and sputum was performed by clinical physicians per the clinical standard operating procedure. Notably, those samples of NPS and BALF for which the viscosity was low required no further sample preparation before FilmArray panel testing, whereas viscous samples, such as sputum, were pre‐treated using the previously described dunk and swirl method.^5^ The remaining specimens were stored in a −80°C freezer for further use.

### 2.3 The FilmArray respiratory panel

In this study, we used version 1.7 of the FA‐RP. It is an automated multiplex PCR detection platform designed to detect 20 common pathogens causing respiratory infection and has a single specimen turnaround time of <2 hours. Tests using the panel were performed following the manufacturer's instructions.

### 2.4 Real‐time RT‐PCR

For specimens that the FA‐RP reported as “Influenza A, not subtyped,” we performed further real‐time RT‐PCR analysis for Influenza A/H7N9, H5N1, and H1N1‐2009 subtyping. A nucleic acid extraction kit (QIAamp Viral RNA Mini Kit, Qiagen) and real‐time PCR kits (Influenza A/B, Influenza A/H1N1‐2009, Avian Influenza A/H7N9, and Avian Influenza A/H5N1 Viral RNA Detection Kits from Fluorescence PCR) were used in accordance with the respective manufacturers’ instructions. For the detection of viral RNA, nucleic acids were extracted from 200‐µL aliquots of each clinical specimen using the QIAamp Viral RNA Mini kit. Individual real‐time PCR assays were performed in 25‐µL volumes in a 7500 real‐time PCR system (Applied Biosystems) using 5 µL of isolated nucleic acid; a universal master mix for RNA (One‐Step RT‐PCR master mix; Zhijiang Biology); universal amplification conditions consisting of one cycle for 10 minutes at 45°C and one cycle for 15 minutes at 95°C, followed by 45 two‐step cycles of 15 seconds at 95°C and 60 seconds at 60°C; and TaqMan fluorogenic chemistry for detection. For all sets of primers and probes, no‐template controls were included in each reaction plate. Specimens that reached the threshold before 35 cycles were considered positive.

### 2.5 Traditional laboratory tests

For both patient groups, traditional standard‐of‐care laboratory diagnostic tests for respiratory infections were performed according to the requests of physicians, including smears, cultures, and serological tests. The serological test in our study was the PNEUMOSLIDE IgM (Vircell) commercial kit based on indirect immunofluorescence.

### 2.6 Evaluation of clinical antimicrobial use

In this study, clinical data, including antimicrobial use, and adjustment or de‐escalation of antimicrobial therapy, were compared between the FA‐RP group and retrospective control group. Turnaround time was defined as the length of time from sample collection to receiving the FA‐RP results. Antimicrobial use was quantified as the defined daily dose (DDD) number, calculated as ∑ (Drug Total Dosage (g)/Drug DDD index).^6^ Adjustment of antimicrobial therapy was defined as a change in antimicrobial regimen immediately after receiving the FA‐RP results.

### 2.7 Statistical analysis and ethics approval

The Mann‐Whitney *U* test was used for the comparison of quantitative data. spss 20.0 (IBM) was used for data analyses. We used graphpad 6.0 for drawing graphs. The Ethics Committee of the Huashan Hospital, Shanghai, approved the study (KY2013‐251; July 2, 2013). All participants gave their written informed consent.

## 3 RESULTS

### 3.1 Population characteristics

**Figure 1:**
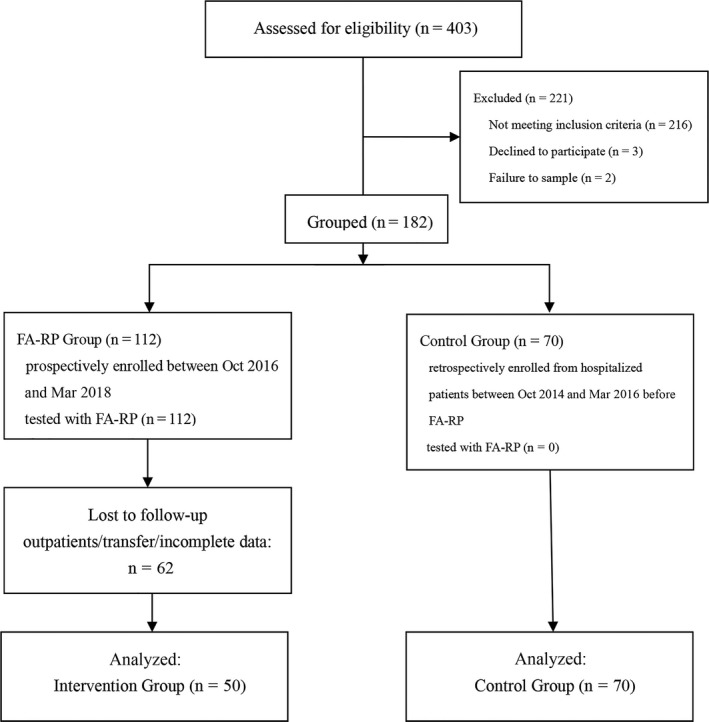
Overview of Patient Enrollment Workflow. A total of 112 patients selected prospectively were submitted to FA‐RP analyses and are referred to as the “FA‐RP group.” Those who were not lost to follow‐up and had complete data are referred to as the “intervention group.” Comparison was made between it and the historical control group

A total of 112 eligible adult patients were prospectively included in the FA‐RP group from October 2016 to March 2018, and samples from all patients were evaluated using the FA‐RP. Among these, 50 patients (44.6%, 50/112) were hospitalized and for whom we had complete clinical data. The remaining 62 patients (55.4%) were lost to follow‐up for outpatients or because of being transferred to other hospitals. As the control group, 70 eligible adult patients were retrospectively enrolled between October 2014 and March 2016 (Figure 1). The baseline characteristics of both patient groups are shown in Table 1.

**Table 1:** Patients’ baseline characteristics

Variable	FA‐RP group (n = 112)	Control group (n = 70)	*P* value
Demographic feature			
Age, mean years ± SD	51.1 ± 17.7	58.6 ± 17.6	.02
Male sex	70 (62.5%)	39 (55.7%)	.25
Clinical feature			
White Blood Cells (×10^9^/L)	7.0 ± 3.1	7.0 ± 3.7	.89
Neutrophil (%)	75.3 ± 14.3	66.4 ± 12.4	<.01
Lymphocyte (%)	15.3 ± 11.3	23.8 ± 10.7	<.01
CRP(mg/L)	56.7 ± 54.3	54.4 ± 50.4	.79
PCT (ng/mL)	0.8 ± 1.7	0.2 ± 0.2	.02
Onset Site			
Community Acquired	75 (67.4%)	45 (64.3%)	.71
Hospital Acquired	37 (32.6%)	25 (35.7%)	

Abbreviations: CRP, C‐reactive protein; PCT, procalcitonin.

### 3.2 Summary of pathogens detected in prospective group patients using the FA‐RP

Among the 112 respiratory samples assessed using the FA‐RP, 86 (76.8%) were positive and 26 (23.2%) were negative. The detected pathogens included Influenza A virus (n = 40, 35.7%), Influenza B (n = 13, 11.6%), *M pneumoniae* (n = 8, 7.1%), adenovirus (n = 8, 7.1%), human rhinovirus/enterovirus (n = 8, 7.1%), coronavirus (n = 5, 4.5%), parainfluenza virus (n = 4, 3.6%), human metapneumovirus (n = 4, 3.6%), and RSV (n = 3, 2.7%; Table 2, Supporting information).

**Table 2:** Pathogens detected by FA‐RP

Pathogens	Cases	Percentage^a^
Influenza A virus	40	35.7%
A H1‐2009	24	
A H3	3	
Not subtyped^b^	13	
Influenza B virus	13	11.6%
Adenovirus	8	7.1%
Enterovirus/Rhinovirus	8	7.1%
Coronavirus	5	4.5%
Coronavirus 229E	3	
Coronavirus HKU1	1	
Coronavirus OC43	1	
RSV	3	2.7%
Parainfluenza virus	4	3.6%
Parainfluenza virus 1	1	
Parainfluenza virus 2	1	
Parainfluenza virus 3	2	
Human Metapneumovirus	4	3.6%
*Mycoplasma pneumoniae*	8	7.1%
Negative^c^	26	23.2%

a Multiple pathogens may be found in one sample; thus, the percentages added up to over 100%.

b When specimens were positive for Influenza A viruses other than H1, H1‐2009, and H3, FA‐RP reported as “not subtyped.”

c Extra microbiological investigations, including smear, culture, or serology, were done for negative specimens. *Pneumocystis jiroveci* (n = 1), *Aspergillus spp.* (n = 1), *Mycoplasma pneumonia* (n = 1), *Mycobacterium tuberculosis* (n = 1), mixed infection with *Enterobacter cloacae*, and *Candida tropicalis* (n = 1) were confirmed.

Mixed infections of multiple pathogens were detected in six patients: Influenza virus/RSV (n = 1), Influenza virus/human metapneumovirus (n = 1), Influenza virus/parainfluenza‐2 (n = 1), *M pneumoniae*/human rhinovirus/enterovirus (n = 1), human rhinovirus/enterovirus/parainfluenza‐2 (n = 1), and adenovirus/coronavirus HKU1/coronavirus OC43 (n = 1).

For 13 samples, the FA‐RP results were “Influenza A/not subtyped.” In these cases, further subtyping was performed using real‐time RT‐PCR. As a consequence, Influenza A virus subtype H7N9 was detected in 11 samples and H1N1‐2009 in the remaining two samples.

### 3.3 Impact on treatment decisions compared with the control group

Among the patients in the FA‐RP group, 53.8% (60/112) received antiviral treatment, including oseltamivir and acyclovir, compared with 12.7% (10/79) in the control group (*P* < .01). Adjustment of antibiotic therapy was recorded for 69.6% (78/112) of the patients in the FA‐RP group, compared with 5.1% (4/79) in the control group (*P* < .01).

As a consequence of the findings of the FA‐FP regarding Influenza A, 15 patients were isolated in a negative pressure ward or single ward, including seven highly pathogenic H7N9 cases. In contrast, in the control group, no comparable isolation measures were undertaken.

When we compared antimicrobial use between the intervention and control groups, we found that the two groups were comparable for the most baseline characteristics (Table 3). Compared with the control group, the intervention group showed no significant decrease of antimicrobial DDDs (control group 14.3 ± 9.6 vs intervention group 13.8 ± 4.6, *P* > .05). However, after excluding antiviral drugs, we detected significantly lower antibiotic/antifungal DDDs (14.1 ± 8.8 vs 13.1 ± 3.3, *P* < .05) for the intervention group. For patients with positive FA‐RP results, both the antimicrobial and antibiotic/antifungal DDDs were significantly lower compared with those in the control group (antimicrobial: 11.5 ± 3.1 vs 14.3 ± 9.6, *P* < .05. antibiotics/antifungals: 10.6 ± 2.5 vs 14.1 ± 8.8, *P* < .01). However, for those patients in the intervention group with negative FA‐RP results, the DDDs were not significantly different from those of control group patients (antimicrobials: 14.1 ± 6.3 vs 14.3 ± 9.6, *P* > .05. antibiotics/antifungals: 13.9 ± 5.1 vs 14.1 ± 8.8, *P* > .05; Table 4).

**Table 3:** Patients’ baseline characteristics

Variable	Intervention group (n = 50)	Control group (n = 70)	*P* value
Demographic feature			
Age, mean years ± SD	53.6 ± 20.7	58.6 ± 17.6	.16
Male sex	33(66.0%)	39(55.7%)	.26
Clinical feature			
Duration (onset to admission)	9.2 ± 4.8	7.0 ± 3.7	<.01
White Blood Cells (×10^9^/L)	6.5 ± 3.5	6.9 ± 2.6	.76
Neutrophil (%)	65.6 ± 24.4	66.4 ± 12.4	.83
Lymphocyte (%)	16.5 ± 13.0	23.8 ± 10.7	<.01
CRP(mg/L)	41.5 ± 48.1	54.4 ± 50.4	.15
PCT (ng/mL)	0.4 ± 1.3	0.17 ± 0.2	.22
Onset site			
Community Acquired	33 (66.0%)	45 (64.6%)	.85
Hospital Acquired	17 (34.0%)	25 (35.4%)	
Pneumonia Severe Index	77.2 ± 33.2	59.9 ± 19.4	<.01

Abbreviations: CRP, C‐reactive protein; PCT, procalcitonin.

**Table 4:** Comparison of antimicrobials’ DDDs between FA‐RP intervention group and control group

	Control group (n = 70)	Intervention group (n = 50)	*P* value	FA‐RP positive (n = 38)	*P* value	FA‐RP negative (n = 12)	*P* value
DDDs^a^							
Antimicrobials	14.3 ± 9.6	13.8 ± 4.6	.13	11.5 ± 3.1	.03	14.1 ± 6.3	.21
Antibiotic/antifungals	14.1 ± 8.8	13.1 ± 3.3	.04	10.6 ± 2.5	<.01	13.9 ± 5.1	.16

a We only calculated DDDs of the first 7 d after admission.

## 4 DISCUSSION

In this study, we applied FA‐RP on diagnosis of UP, which is a certain type of pneumonia causing mainly by viruses and atypical pathogens. We found that the FA‐RP detected pathogens in 76.8% (86/112) of all specimens, which is higher than that the detection rate obtained when this technique was applied for general community‐acquired pneumonia (38.6%, 49/127).^7^ These results, therefore, show that when applied clinically for UP, the FA‐RP would provide a higher probability of identifying causal pathogenic microorganisms.

The findings of the present study also confirmed that sputum and BALF could be used as samples for FA‐RP tests, which contrasts with the manufacture's assertion that NPS stored in viral transport media (VTM) is the only suitable material that is compatible. Indeed, we obtained positive FA‐RP results for all the types of respiratory tract specimens. Notably, however, when performing this test, pre‐treatment of specimens with high viscosity, such as sputum, is essential; otherwise, the reactions are likely to fail. Here, we pre‐treated samples following the procedure described by Branche,^5^ using a clean swab to stir the sputum and then placing it into VTM, so that the pathogens were transferred into a low viscosity medium that is suitable for the FA‐RP. Previous studies have reported that lower respiratory tract specimens should be accorded higher sampling priority (ie BALF > sputum > NPS), and have indicated that the performance of the FA‐RP in patients with pneumonia could be improved by using BALF.^8^, ^9^, ^10^ Consistently, in the present study, we obtained higher positive rates when using BALF and sputum samples than when using NPS, although the difference was not significant (78.8% vs 61.5%, *P* = .166). Moreover, in recent years, saliva has also shown potential as a suitable material for the molecular detection of respiratory viruses.^11^

We prospectively enrolled patients diagnosed with UP during the period from October 2016 to March 2018, and attempted to identify the causal pathogens. We accordingly found that Influenza A virus was most commonly reported causal agent, followed by Influenza B virus, adenovirus, rhinovirus/enteric virus, etc Other studies on adult viral pneumonia have reported similar patterns of pathogen distribution,^12^, ^13^ with Influenza viruses being the predominant causal agents, although the proportion of Influenza viruses detected in the present study was notably higher (47.3% vs 8%).

It is worth noting that for the commercial FA‐RP used in the present study, identification of Influenza A virus is limited to the H3, H1, and 2009‐H1 subtypes, whereas for other subtypes, such as H7N9, the FA‐RP can only provide the vague result of “not subtyped Influenza A.” However, in mainland China, Influenza A virus H7N9 is one of the epidemic strains associated with UP,^14^ and indeed, this strain was particularly prevalent during the winter and spring of 2016‐2017. Compared with other Influenza A infection, H7N9 patients tend to present with a more clinically severe manifestation and require more aggressive treatment. Thus, in these cases, further subtyping is necessary. In the study, we obtained test results of “Influenza A virus not subtyped” in 13 cases, among which 11 were finally identified as H7N9 based on real‐time PCR. Consequently, in clinical practice, when FA‐RP reports a not subtyped Influenza A virus, further subtyping, particularly for avian influenza virus, including H7N9, H5N1, and H5N6 should be carried out immediately.

The findings of the present study show that the FA‐RP offers a relatively short turnaround time compared with traditional methods. The median turnaround time, from sampling to reporting the results to physicians, was 1.6 hours, compared with that of culturing (more than 48 hours) and serum IgM tests (more than 4 hours). This feature of the FA‐RP may be of particular benefit with respect to the clinical management of pneumonia of unknown cause.

In the present study, we found that 53.8% (60/112) of the patients in the FA‐RP group received antiviral treatment, which is significantly higher than that in the control group (14.3%, 10/70; *P* < .001). Among those patients who received a positive test result for the Influenza virus, 100% (43/43) were treated with oseltamivir that is notably higher than the 74%‐81% reported in other studies carried out in the United States.^15^, ^16^ Moreover, adjustment of antibiotic therapy was reported for 69.6% (78/112) of the patients in the FA‐RP group, compared with only 5.1% (4/79) in the control group. These findings strongly indicate that the results of the FA‐RP test may assist in clinical decision making, particularly with respect to reducing unnecessary antibiotic usage in the treatment of UP.

The rapid identification of pathogens causing respiratory tract infection has both essential clinical and public health values. In the present study, 15 of the enrolled patients were promptly isolated as soon as the diagnosis of Influenza virus infection had been established. In contrast, for the control group patients who did not receive precise diagnosis of causing agents, no comparable isolation measures were undertaken. Such a lack of an accurate diagnosis may lead to a delay in patient isolation and, consequently, the wider spread of viruses. Moreover, for these patients, we consciously sought to regularly analyze the viral nucleic acids in the patient's respiratory specimens using real‐time PCR, and patients were discharged only after the nucleic acid test proved negative, thereby reducing the risk of the disease spreading.

To further evaluate the clinical impact of the FA‐RP, we compared the antibiotic use of adult UP patients in the FA‐RP cohort and the historical control cohort, the latter of which were diagnosed only by using conventional methods. We found that compared with the control cohort, the intervention group had significantly lower antimicrobial DDDs, particularly with respect to the use of antibiotics and antifungal drugs. These results thus serve to highlight that given the benefit of an accurate diagnosis of the etiology, a more reasonable antibiotic usage could be achieved. These results are partially consistent with the findings of some previously published studies. For example, Rogers et al^17^ evaluated the outcomes in pediatric patients (3 months to 21 years old) admitted to hospital with an acute respiratory illness, and, similar to the findings of the present study, found that the use of FA‐RP facilitated a reduction in the duration of antibiotic use and the length of hospitalization. However, another randomized controlled trial discovered that use of the FA‐RP was not associated with a reduction in the overall duration of antibiotic usage.^18^ Nevertheless, a higher number of patients in the FA‐RP group received single doses or brief courses of antibiotics than those in the control group. Given these inconclusive findings, further studies are required concerning the impact of FA‐RP on the clinical decisions regarding pneumonia.

We compared the duration of hospital stay between the two groups. As a result, intervention group unexpectedly showed longer duration of hospital stay than the control group (12.1 vs 7.6 days, *P* = .001). One reasonable explanation is that subjects in the intervention group had more severe cases of pneumonia than those in the control group (higher PSI, Table 3), that is, a more severe condition may lead to a prolonged hospital stay. More importantly, stricter criteria of discharge were required for the intervention group. According to regulations sanctioned by the Chinese Ministry of Public Health in 2009, discharge of Influenza A H1N1 patients demands both a recovery of condition and two consecutive negative test results of viral nucleic acid in respiratory specimens. Therefore, intervention group patients positive for Influenza A took follow‐up PCR tests. The patients could not be discharged from the hospital until they met the criteria. In contrast, for the control group, lacking precise diagnosis of causing agents, no such criteria were required. In our practice, application of FA‐RP prolonged the length of stay; however, we believe additional benefits concerning public health and disease control were achieved.

This present study is one of the several that have examined the application of FA‐RP in Asia.^19^, ^20^, ^21^, ^22^, ^23^, ^24^ Compared with other published studies, our study focused on adults with more severe pneumonia conditions, and to the best of our knowledge, it is the only study that has covered two consecutive pandemic seasons. More importantly, it describes the positive clinical impacts of the FA‐RP, particularly in terms of antibiotic use. Similarly, Duan et al^24^ examined the use of the FA‐RP in the management of lower respiratory tract infections in a larger population in Beijing. A shorter length of hospitalization and a reduction in antibiotic use were observed. Other studies, however, tended to focus on the performance of the FA‐RP or merely a description of the detected pathogens.

Our study does, however, have a few limitations. Notably, the sample size was limited, and the study was conducted only at a single location, which may thus limit the general applicability of the findings. In addition, the study included two cohorts from different time periods; hence, there may have been differences in factors that influenced clinician behavior between the two periods that were not accounted in our analysis. Furthermore, the incidence of various respiratory viruses may have varied across the two periods that may thus limit the validity of a direct comparison of clinical outcomes.

In conclusion, the FA‐RP is a rapid and sensitive NAAT method that can be used for the diagnosis of UP in adults. In the two recent years in Shanghai, the most common pathogen causing UP was identified as the Influenza virus. However, in this regard, it should be taken into consideration that the current version of the FA‐RP is unable to directly detect H7N9. Nevertheless, the results obtained in this study show that the FA‐RP may make a valuable contribution to clinical decision making and facilitate the reasonable use of antibiotics.
