# Supplementary material for: Rapid detection of respiratory organisms with FilmArray respiratory panel and its impact on clinical decisions in Shanghai, China, 2016‐2018
Source: Influenza Other Respir Viruses. 2019 Dec 1;14(2):142–9. doi: 10.1111/irv.12701 (PMC7040966; doi:10.1111/irv.12701)
Supplement: Supplementary file 1 [file IRV-14-142-s001.docx]

Supplementary Information

1. Time-Series Data of FA-RP.

Between Oct 2016 and Mar 2018, there were two high-occurrence seasons for UP, Jan-Mar, 2017 and Jan-Mar, 2018 with the most cases collected (Figure S1), and this is in accordance with the epidemiology of pneumonia. During the first epidemic season (Jan-Mar, 2017), 8 out of 27 samples (29.6%) reported H7N9, 3/27 (11.1%) Mycoplasma pneumonia, 1/27 (3.7%) A H3, 1/27 (3.7%) metapneumovirus. During the second epidemic season (Jan-Mar, 2018), H1-2009 predominated with 26 out of 45 samples (57.7%), followed by influenza B (13/45, 28.9%), metapneumovirus (2/45,4.4%), parainfluenza virus (1/45, 2.2%). During other periods, occurrence of UP was much lower, and pathogens detected were sporadic rather than epidemic throughout time. For example, during Oct-Dec, 2017, only 15 cases of UP were collected, and FA-RP detected coronavirus (n=3, 23.1%), rhinovirus/enterovirus(n=3, 23.1%), adenovirus(n=2, 15.4%), Mycoplasma pneumonia (n=2, 15.4%), and parainfluenza virus (n=1, 7.7%) respectively.

This study showed that pathogens causing UP would change with the general epidemic trend. During Jan-Mar 2017, most cases of influenza virus were influenza A virus H7N9, a small proportion being H3. but during Jan-Mar 2018, influenza A virus 2009-H1 and influenza virus B were only two subtypes detected. This is in accordance with influenza epidemic data in China in the last 2 years. The winter season in 2016-2017 was called the fifth peak of influenza A H7N9, while in the winter of 2017-2018, according to WHO data, subtypes of influenza epidemic in mainland China were 2009-H1N1 and influenza B virus, with a small amount of H3 virus, same as our results.

**Figure S1.** Time-Series Data of FA-RP.

Time-Series data of FA-RP results. In two Influenza epidemics, Influenza A/H7N9, Influenza A/H1-2009 and Influenza B predominated the pathogen distribution. During other seasons, pathogens were sporadic through time. RSV= Respiratory Syncytial Virus, PIV= Parainfluenza, MP= Mycoplasma pneumonia, hMPV= Human Metapneumovirus, Flu B= Influenza B, A H3= Influenza A/H3, A H7N9= Influenza A/H7N9, A H1-2009= Influenza A/H1-2009, EV/RV= Human Rhinovirus/Enterovirus, COV= Coronavirus, Adv= Adenovirus, Neg= Negative.

1. Comparison of results between FA-RP and serology

The serological test used in our study was the PNEUMOSLIDE IgM (Vircell, Spain) commercial kit based on indirect immunofluorescence. It’s able to detect IgM antibody for nine common respiratory pathogens from serum, including influenza A/B, respiratory syncytial virus (RSV), adenovirus, parainfluenza (type 1, 2, 3), *Chlamydophila pneumonia, Legionella pneumophila, Rickettsia burneti and Mycoplasma pneumonia.*

A total of 41 patients underwent both FA-RP and serology tests at the same time. Serum IgM tests were negative in 20 out of 21 cases with positive FA-RP results, including 12 influenza virus A, 2 influenza B, 1 parainfluenza, 2 respiratory syncytial virus and 4 adenovirus. However, for 5 cases of Mycoplasma pneumonia detected by FA-RP, all of them also had positive serum IgM test results (Table S1).

Table S1. Comparison between FA-RP and Serology^†^

|  | FA-RP+/  Ser+^‡^ | FA-RP+/  Ser-^§^ | FA-RP-/  Ser+ | FA-RP-/  Ser- |
| --- | --- | --- | --- | --- |
| Influenza A | 1 | 11 | 0 | 29 |
| Influenza B | 0 | 2 | 1 | 38 |
| Parainfluenza | 0 | 1 | 0 | 40 |
| Respiratory Syncytial Virus | 0 | 2 | 0 | 39 |
| Adenovirus | 0 | 4 | 0 | 37 |
| Mycoplasma pneumonia | 5 | 0 | 2 | 34 |

†. This table compared results of FA-RP and serology tests in 41 patients who underwent both methods. FA-RP detected more viruses than serology (FA-RP+/Ser-). For Mycoplasma pneumonia, two methods shared a high accordance. Only pathogens within both methods’ detection range were compared.

‡. Number of cases that were both FA-RP and serology positive. Ser: Serology.

§. Number of cases that were FA-RP positve while serology negative, etc.

1. Comparison of results between FA-RP and culture

Among the 112 patients, samples from 75 patients were also assessed via microbiological culture using synchronous respiratory specimens. Among these, 56 were FA-RP positive, whereas 28 were culture positive. However, in only two cases and one case were bacteria and fungi, respectively, considered causative pathogens. Finally, one case of mixed infection (*Candida glabrata* determined by culturing and influenza B determined using the FA-RP), two cases of bacterial/fungal infection (*Enterococcus faecium* and *Aspergillus fumigatus*, separately, both FA-RP negative), and 55 cases of single virus infection were clinically diagnosed.
